# Patient-reported outcomes of parenteral somatostatin analogue injections in 195 patients with acromegaly

**DOI:** 10.1530/EJE-15-1042

**Published:** 2016-03

**Authors:** Christian J Strasburger, Niki Karavitaki, Sylvère Störmann, Peter J Trainer, Ilonka Kreitschmann-Andermahr, Michael Droste, Márta Korbonits, Berit Feldmann, Kathrin Zopf, Violet Fazal Sanderson, David Schwicker, Dana Gelbaum, Asi Haviv, Martin Bidlingmaier, Nienke R Biermasz

**Affiliations:** 1 Department of Medicine for Endocrinology, Diabetes and Nutritional Medicine, Charité Universitätsmedizin, Campus Mitte, Charitéplatz 110117, Berlin, Germany; 1 Oxford Centre for Diabetes, Endocrinology and Metabolism, Oxford, UK; 2 Medizinische Klinik und Poliklinik IV, Klinikum der Universität München, Munich, Germany; 3 Department of Endocrinology, The Christie, Manchester, UK; 4 Department of Neurosurgery, University of Erlangen Nuremberg, Erlangen, Germany; 5 Department of Neurosurgery, University of Duisburg-Essen, Essen, Germany; 6 Practice for Endocrinology, Oldenburg, Germany; 7 Endocrinology, Barts and the London School of Medicine, Queen Mary University, London, UK; 8 Endokrinologie and Diabetologie im Zentrum, Stuttgart, Germany; 9 Phase IV Programs, Basle, Switzerland; 10 Chiasma, Newton, Massachusetts, USA; 11 Endocrine Research Laboratories, Medizinische Klinik und Poliklinik IV, Klinikum der Universität München, Munich, Germany; 12 Leiden University Medical Centre, Leiden, The Netherlands

## Abstract

**Background:**

Long-acting somatostatin analogues delivered parenterally are the most widely used medical treatment in acromegaly. This patient-reported outcomes survey was designed to assess the impact of chronic injections on subjects with acromegaly.

**Methods:**

The survey was conducted in nine pituitary centres in Germany, UK and The Netherlands. The questionnaire was developed by endocrinologists and covered aspects of acromegaly symptoms, injection-related manifestations, emotional and daily life impact, treatment satisfaction and unmet medical needs.

**Results:**

In total, 195 patients participated, of which 112 (57%) were on octreotide (Sandostatin LAR) and 83 (43%) on lanreotide (Somatuline Depot). The majority (>70%) of patients reported acromegaly symptoms despite treatment. A total of 52% of patients reported that their symptoms worsen towards the end of the dosing interval. Administration site pain lasting up to a week following injection was the most frequently reported injection-related symptom (70% of patients). Other injection site reactions included nodules (38%), swelling (28%), bruising (16%), scar tissue (8%) and inflammation (7%). Injection burden was similar between octreotide and lanreotide. Only a minority of patients received injections at home (17%) and 5% were self-injecting. Over a third of patients indicated a feeling of loss of independence due to the injections, and 16% reported repeated work loss days. Despite the physical, emotional and daily life impact of injections, patients were satisfied with their treatment, yet reported that modifications that would offer major improvement over current care would be ‘avoiding injections’ and ‘better symptom control’.

**Conclusion:**

Lifelong injections of long-acting somatostatin analogues have significant burden on the functioning, well-being and daily lives of patients with acromegaly.

## Introduction

Acromegaly is a rare debilitating endocrine disease characterised by hypersecretion of growth hormone (GH), occurring almost exclusively as a result of a benign pituitary adenoma (1).

The disease is associated with significantly increased morbidity, mortality, mainly due to cardiovascular, cerebrovascular and respiratory diseases and decreased quality of life (1, 2, 3).

Somatostatin receptor ligands ((SRLs): octreotide and lanreotide) are used as first-line medical treatment for patients with inadequate response to surgery and/or radiotherapy, or for those in whom surgery and/or radiotherapy are not indicated (4, 5, 6, 7, 8, 9, 10). These are long-acting depot formulations administered as monthly intramuscular or deep subcutaneous injection (7, 10).

Published literature on patient-reported outcomes (PROs) in acromegaly is scarce. Quality of life and illness perception studies have been mainly performed in cross-sectional settings, in which small group sizes and heterogeneity of the population studied precluded strong conclusions on outcome of a specific treatment (11, 12). Next, studies have focused on acromegaly symptoms or disease-specific quality of life without specifically addressing the impact of chronic parenteral injections (13). To date, no qualified questionnaire is available addressing these issues. This PRO survey was designed to comprehensively assess for the first time the impact of chronic administration of long-acting SRL injections on the physical and psychological well-being and the everyday lives of patients with acromegaly (11, 12, 13).

## Patients and methods

### Survey design

This was a multi-centre, observational survey, in 195 patients with acromegaly managed routinely with parenteral SRLs (14). The survey protocol was developed with internationally recognised experts in endocrinology, experienced in the care of patients with acromegaly with medical treatment, and received approval by local Ethics Committees in eight endocrinology centres and one neurosurgical centre (Erlangen), specialised in acromegaly in three European countries, Germany, UK and The Netherlands.

Participation was suggested to all acromegaly patients treated by the participating sites. Patients were eligible for participation if they were above 18, diagnosed with acromegaly, treated with regular parenteral SRL injections for a minimum of 6 months and able to successfully complete an interview and independently respond to the questions. The survey questionnaire was administered after a written informed consent was obtained, and data collected were pseudonymised for confidentiality.

Retrospective clinical information including acromegaly disease history, IGF1 assays, serum IGF1 levels and SRL treatment history were collected from the patients' medical files. The treating physician was asked to evaluate the patients' response to treatment. The patient questionnaire was developed to capture the impact and burden of chronic parenteral injections as well as the patient's satisfaction with treatment.

This questionnaire is composed of five major sections: i) acromegaly signs and symptoms since the last injection (13 items), graded by severity from none (0) to severe (3) and their impact on patients (four statements); ii) injection-related physical burden, based on patients' overall historical experience with SRLs treatment (19 items plus two statements) (graded by burden from 0=none/not troublesome to 3=very troublesome); iii) injection-related emotional burden (seven statements) (graded by frequency from 0=not at all to 3= often); iv) injection-related everyday life burden throughout the entire treatment experience (12 items); and v) overall treatment satisfaction (two items) and unmet medical needs (15 yes/no statements).

The questionnaire was interviewer administered by specifically trained research nurses at the sites either by telephone or in person and took ∼30 min to complete. Patients' responses were captured ‘as is’ with no amendments or interpretations by the interviewer.

### Statistical considerations and data analysis

This observational survey is descriptive in nature (14). No formal hypothesis testing was used (15, 16). In total, 195 participants provided a sample size clinically sufficient to represent real-life population of acromegaly patients in the selected sites and to show trends for the burden and/or satisfaction with chronic injections in this rare disease. This sample size is aligned with other observational studies in acromegaly (17, 18). A formal analysis plan predefined the analyses populations and the descriptive variables to be assessed (15).

*Post hoc* exploratory analysis (19) was employed to analyse differences between the two SRLs octreotide and lanreotide, between biochemically controlled and non-controlled patients (age-normalised IGF1 SDS ≤2.0; >2.0) as well as patients with partially controlled and non-controlled disease (SDS ≤2.6; >2.6).

Statistical analysis was conducted between groups with the unequal variance (uv) *t* test (20). Missing data were not imputed, hence not included in the analysis (16, 20).

To allow comparison across different laboratories, all IGF1 values were translated into SDS using assay-specific reference intervals stratified by age and sex, as recently published and recommended in the international consensus criteria (21). To that purpose, recently published reference intervals and the formula *z*=(((IGF1/*M*)^*L*^)-1)/(L×S)) were used for IGF1 values generated on the IDS-iSYS assay (22). For the two laboratories using the Immulite platform, SDS was calculated based on the respective reference intervals and the formula *z*=(log IGF1-log mean)/logSD (23).

### Patient population

A total of 301 acromegaly patients were invited to participate between November 2012 and June 2013. In total, 201 patients signed an informed consent, five patients were lost to follow-up and one patient was excluded from the analysis due to incomplete SRL treatment data. The 195 eligible patients were distributed between Germany (five sites), 102 patients; UK (three sites), 70 patients; and The Netherlands (one site), 23 participants. Table 1 summarises the baseline characteristics of the enrolled population.

In total, 112 patients (57.4%) were receiving octreotide while 83 patients (42.6%) were receiving lanreotide. The most commonly prescribed administration frequency was every 28 days in 138 patients (69.7%), while in 28 patients (13.8%), this was shorter (21 days), and in 11.3% of patients, this was longer (14 patients – 42 days and eight patients – 56 days). The mean treatment duration with SRLs in the enrolled population was 6.6 years (range 0.5–18.2 years). In total, 42 patients (21.1%) received dopamine agonists and 33 patients (16.6%) pegvisomant, in combination with SRLs. A total of 70 patients (36%) had been switched to their current treatment from a different medical treatment for acromegaly: from dopamine agonists to SRLs (20 of 70) and between SRLs (octreotide to lanreotide (19 out of 70), lanreotide to octreotide (three out of 70), daily s.c. octreotide to long-acting SRLs (three out of 70) and data not provided (25 patients)). The most common reason for a switch was a lack of disease control (49%). Other reasons to switch were newly available treatments (16%), adverse reactions (11%) and injection-related symptoms/signs (7%).

## Results

### Acromegaly signs and symptoms

**Figure 1:**
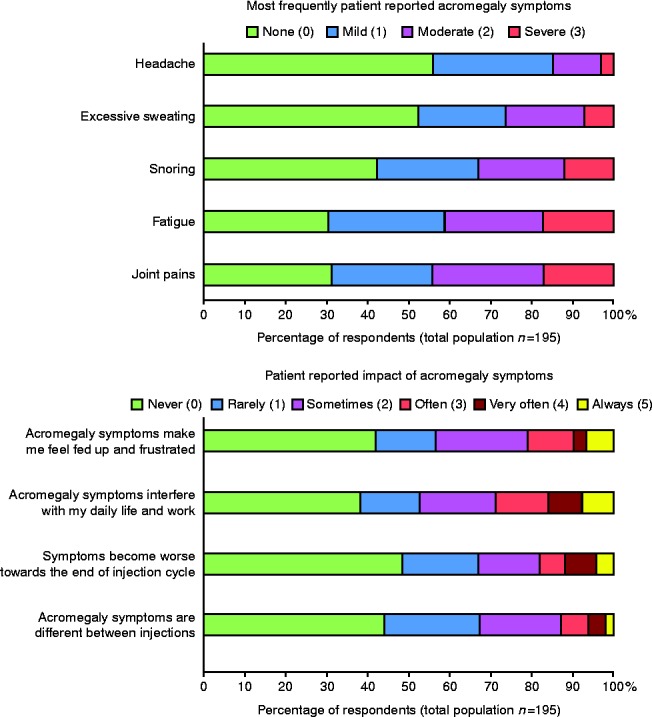
Acromegaly symptoms and their impact on patients.

The majority of patients (>70%) reported acromegaly symptoms, with fatigue, joint pains, snoring, excessive sweating and headaches being the most frequently described. In total, 52% of all patients reported that their symptoms worsened towards the end of the dosing interval and 62% that symptoms interfered with their daily life (Fig. 1).

There was no statistically significant difference in the mean acromegaly symptom scores between biochemically controlled or partially controlled and uncontrolled patients (Supplementary Tables 4 and 5, see section on supplementary data given at the end of this article).

### Injection-related signs and symptoms

**Figure 2:**
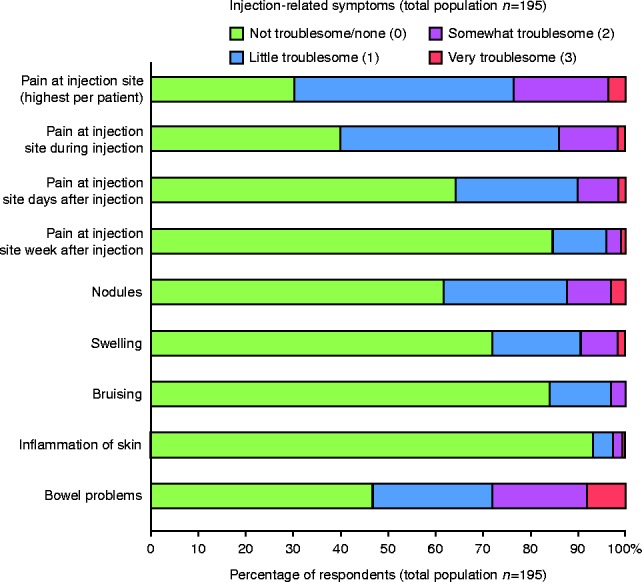
Injection-related symptoms (total population; *n*=195).

The major injection-related signs and symptoms reported by the patients are summarised in Fig. 2 and provided in detail in Supplementary Tables 1, 2, and 3, see section on supplementary data given at the end of this article.

Injection-related pain was the item scored highest by patients, with a mean severity burden of 0.97 (s.d.=0.80), followed by bowel problems (mean=0.90; s.d.=0.99). Hardness at the injection site was the highest scored skin pathology (mean=0.64; s.d.=0.78), followed by nodules (mean=0.54; s.d.=0.79). More than a third (36%) of patients reported pain for days after their injection and 15% for a week after their injection.

**Figure 3:**
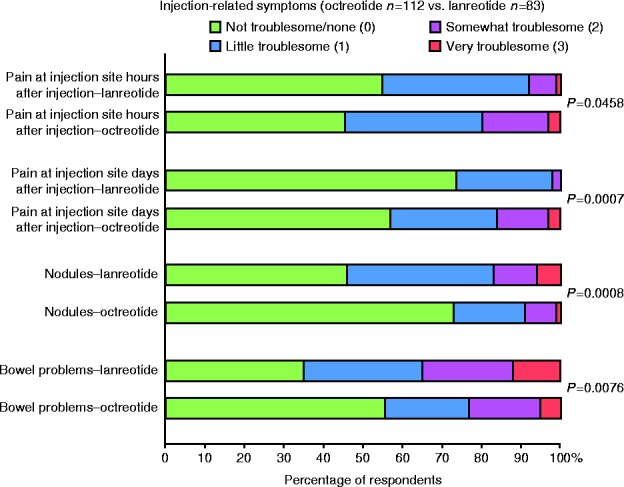
Injection-related symptoms (octreotide and lanreotide populations).

Overall, the treatment burden for octreotide and lanreotide patients was similar. However, patients on octreotide reported pain for a longer duration after injections. On the other hand, patients treated with lanreotide tended to develop more skin nodules, swelling, bruising and dermatitis compared with those treated with octreotide, as well as more bowel problems (Fig. 3).

### Emotional impact

**Figure 4:**
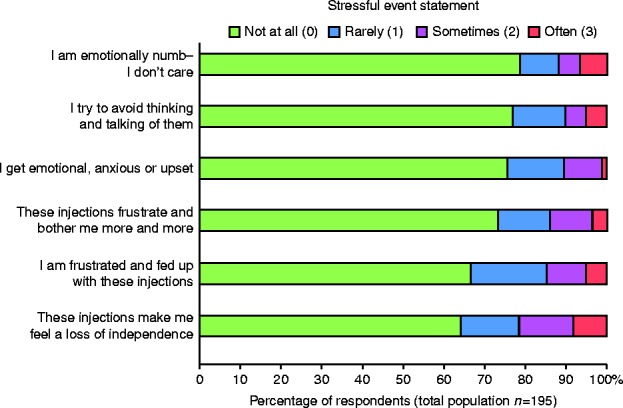
Emotional impact of the injections.

Figure 4 shows that one-third of patients in the survey group felt emotionally burdened by the injections, with 36% of them reporting a ‘loss of independence’ as a consequence of their treatment.

### Everyday life impact

The majority of patients received their SRL injections at their general practitioner's (GP's) office or hospital outpatient clinic (68%), and in most cases, these were administered by the nurse or the physician (39 and 33% respectively). This was remarkably similar between the treatments, and only 17% of patients (16% of octreotide and 18% of lanreotide-receiving patients) reported that they received the injections at home. Only eight patients (10%) treated with lanreotide reported they were self-injecting their medication (2% on octreotide). Germany, UK and The Netherlands showed notable similarity regarding the above treatment patterns.

Transportation to and from the GP's office or clinic to receive injections was considered convenient or very convenient by 62% of the survey population, while 22% of patients considered the travel as somewhat or very inconvenient. In total, 7% of participants were routinely accompanied by an additional person. The total time required for the injections, including travel; waiting; preparation; and administration of the injection, consultation and arranging the next appointment, was reported as 67 min (range 0 (for self-injection) – 300 min; s.d. 48 min) per injection on average.

Work loss due to the injections was reported by 16.6% of the population, with mean of 11 times/year.

In total, 44% of the patients had encountered problems with the preparation and administration of the injections at any time during their SRL treatment history, with an average of 7.6 (range 1–50; s.d.=9.3) problems per patient (during a mean of 6.6 years of treatment). The majority of these cases (42%) were clogged or broken needles, often necessitating a second injection (27% of all consequences).

### Patients' satisfaction and unmet medical needs

**Figure 5:**
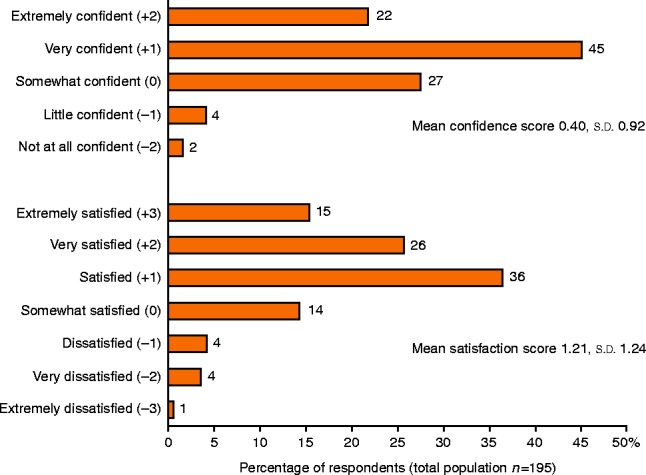
Patient overall satisfaction and confidence.

Patients' satisfaction with their SRL treatment is summarised in Fig. 5. Patients were generally satisfied with their treatment, yet reported that an ‘oral therapy’ (48%), ‘a treatment to avoid injections’ (44%) and a ‘treatment with better symptom control’ (41%) would be potential major improvements over their current treatment. Self-injection or at-home injection was not considered as a major improvement by many patients (12 and 10% respectively). There was no statistically significant difference between biochemically controlled or partially controlled and uncontrolled patients (Supplementary Tables 4 and 5).

## Discussion

The high participation rate in this survey (201 enrolled out of a 301 eligible patients) suggests that this survey represents a real-world population of patients with acromegaly treated with SRLs at the respective participating endocrinology centres in Europe. Treatment and disease characteristics are similar to clinical trials and registries data in the literature, except for a higher age and a lower biochemical control rate (SDS ≤2.0; 36.4%) (2, 4, 7, 8, 10, 13, 17). This may reflect differences between real-world practices and randomised controlled trials and is also consistent with recently published data (13, 18). The majority of patients (>70%) reported symptoms consistent with acromegaly despite treatment. The prevalence of reported acromegaly symptoms was higher in this cohort as compared with clinical trial literature (4, 7, 8, 10), yet is comparable to that reported in the observational SODA registry (17).

While the physical burden of chronic injections as reported in this survey is consistent with the literature (4, 5, 6, 7, 8, 9, 10), the incidence of injection site reactions is distinctly higher, specifically for injection site pain, nodules, swelling or bruising. The longer duration of pain following octreotide injections and higher incidence of skin pathologies, particularly nodule formation, on lanreotide, is interesting, reported here for the first time, and reflects clinical practice experience. Injection patterns were similar between octreotide and lanreotide (including the incidence of home injections), which suggest that home or self-injection rates are generally low in the participating European countries and are considerably lower than reported for the USA (SODA) (17, 24).

The similarities in the reported issues encountered during injections (e.g. high frequency of clogged needles), in different countries imply that these problems are inherent to the medications themselves and their mode of administration and less so affected by the standard of care in a given country. Time loss due to injections (on average over 0100 h/injection) is corroborated with the literature (10, 17).

The majority of patients (>70%) reported acromegaly symptoms, with fatigue, joint pains, snoring, excessive sweating and headaches being the most frequently described. An unexpected finding was that half (52%) of the participants reported that their symptoms became worse towards the end of the dosing interval (‘breakthrough symptoms’). Based on these results, it may be speculated that the shortened injection interval reported by some patients could have been due to breakthrough symptoms rather than to inadequate control of IGF1 levels. Our data show for the very first time that breakthrough symptoms reported by patients are a considerable treatment burden in general practice and deserve the attention of the treating physician.

*Post hoc* exploratory analyses detected no significant differences between biochemically controlled and non-controlled patients (SDS ≤2.0; >2.0) as well as patients partially controlled and non-controlled (SDS ≤2.6; >2.6), with regards to patient-reported symptoms burden, physical and emotional burden or overall treatment satisfaction. No correlation was noted between the objective biochemical parameter IGF1, the main treatment focus and the subjective sense of well-being reported by patients enrolled in this survey, which are regularly managed with SRLs. This is in keeping with previous published data (11, 12, 13, 18).

Despite the considerable treatment burden noted with chronic injections, patients were both satisfied with their treatment and confident that it provided benefit to them. This is not surprising, as SRL injections provide an effective treatment (4, 7, 8, 10, 24). Patients still reported that potential major improvements over their current treatment would be one that would avoid injections and a treatment with better symptom control.

This PRO survey has some limitations. It is an uncontrolled, observational survey that provides a snapshot in time on PROs related to treatment with SRLs (14). As such, causative effects on outcomes cannot be determined (14, 19). Assessing PROs in acromegaly patients treated with other medical treatments may shed more light on the etiology of symptoms and their possible mediation by IGF1 effects vs GH effects. Assessing PROs in patients with no functioning adenoma may discriminate between non-specific symptoms related to the tumour location/burden and symptoms related to excess GH. The survey questionnaire used in our present study was specifically designed to investigate the burden of monthly SRL injections (injection site reactions, breakthrough symptoms, day-to-day burden of chronic injections provided by health care providers in the clinic and other). New survey questionnaires could be developed for use in future studies that are specific to other treatments and/or indications and may help to better understand the etiology of symptoms experienced by acromegaly patients.

Despite the broad inclusion criteria and a high participation rate, an enrolment bias towards more burdened acromegaly patients cannot be excluded, given the relatively low rate of biochemical control and the frequency of symptoms reported, although, as suggested previously, this may reflect the real-world population in contrast to clinical trials. In addition, patients were recruited and interviewed by the research nurses at specialised endocrinology centres, with whom patients had established good and long-standing relationships. This may have led to an underreporting bias.

These novel patient-reported data, which were evaluated for the first time in such comprehensive fashion, reflect the way that chronic injections of long-acting SRLs impact the functioning, well-being and daily lives of patients with acromegaly. These effects are substantial and remarkably consistent across both octreotide and lanreotide patients and in the three European countries studied. It appears that variation of symptom control throughout the treatment interval is not a negligible issue that has not yet been addressed in studies primarily focusing on efficacy. Future studies should further explore the clinical significance of breakthrough symptoms and potential strategies to improve stability of control.

In parallel to biochemical disease control, treating physicians and future clinical studies should consider assessment of PRO measures, as clinical symptoms and patients' well-being. PRO studies such as the current survey highlight the need for better therapeutic alternatives for optimal control of acromegaly.

## Supplementary data

This is linked to the online version of the paper at http://dx.doi.org/10.1530/EJE-15-1042.

## Figures and Tables

**Table 1:** Baseline characteristics.

**Patients' characteristics**	***n***	**%**
Gender		
Male (*n*/%)	103	52.8
Female (*n*/%)	92	47.2
Age (based on year of birth)		
Mean (years)	59	
Minimum (years)	24	
Maximum (years)	89	
Median (years)	61	
s.d.	14.0	
Duration of disease		
<10 years (*n*/%)	99	50.8
Between 10 and 20 years (*n*/%)	61	31.3
>20 years (*n*/%)	35	17.9
Unknown (*n*/%)	0	0.0
Physician assessment of response to SSA treatment		
Responder (*n*/%)	104	53.3
Partial responder (*n*/%)	71	36.4
Non-responder (*n*/%)	12	6.2
Missing information	8	4.1
IGF1 (*n*)	187	
Mean (ng/ml)	244	
Minimum (ng/ml)	45	
Maximum (ng/ml)	1224	
Median (ng/ml)	202	
s.d.	150	
Missing information (*n*)	8	
Biochemical control (*n*) (SDS refers to IGF1)		
Controlled (SDS ≤2.0)	71	36.4
Not controlled (SDS >2.0)	109	55.9
Controlled plus ‘partially’ controlled (SDS ≤2.6)	101	51.8
Missing information	15	7.7
Drug and dosage		
Octreotide (*n*/%)	112	57.4
10 mg (*n*/%)	16	8.2
20 mg (*n*/%)	28	14.4
30 mg (*n*/%)	68	34.9
Lanreotide (*n*/%)	83	42.6
60 mg (*n*/%)	9	4.6
90 mg (*n*/%)	22	11.3
120 mg (*n*/%)	52	26.7
